# Small Molecule Deubiquitinase Inhibitors Promote Macrophage Anti-Infective Capacity

**DOI:** 10.1371/journal.pone.0104096

**Published:** 2014-08-05

**Authors:** Marie-Eve Charbonneau, Marta J. Gonzalez-Hernandez, Hollis D. Showalter, Nicholas J. Donato, Christiane E. Wobus, Mary X. D. O’Riordan

**Affiliations:** 1 Department of Microbiology and Immunology, University of Michigan, Ann Arbor, Michigan, United States of America; 2 Department of Internal Medicine, University of Michigan, Ann Arbor, Michigan, United States of America; 3 Vahlteich Medicinal Chemistry Core, Department of Medicinal Chemistry, College of Pharmacy, University of Michigan, Ann Arbor, Michigan, United States of America; University of Illinois at Chicago College of Medicine, United States of America

## Abstract

The global spread of anti-microbial resistance requires urgent attention, and diverse alternative strategies have been suggested to address this public health concern. Host-directed immunomodulatory therapies represent one approach that could reduce selection for resistant bacterial strains. Recently, the small molecule deubiquitinase inhibitor WP1130 was reported as a potential anti-infective drug against important human food-borne pathogens, notably *Listeria monocytogenes* and noroviruses. Utilization of WP1130 itself is limited due to poor solubility, but given the potential of this new compound, we initiated an iterative rational design approach to synthesize new derivatives with increased solubility that retained anti-infective activity. Here, we test a small library of novel synthetic molecules based on the structure of the parent compound, WP1130, for anti-infective activity *in vitro*. Our studies identify a promising candidate, compound 9, which reduced intracellular growth of *L. monocytogenes* at concentrations that caused minimal cellular toxicity. Compound 9 itself had no bactericidal activity and only modestly slowed *Listeria* growth rate in liquid broth culture, suggesting that this drug acts as an anti-infective compound by modulating host-cell function. Moreover, this new compound also showed anti-infective activity against murine norovirus (MNV-1) and human norovirus, using the Norwalk virus replicon system. This small molecule inhibitor may provide a chemical platform for further development of therapeutic deubiquitinase inhibitors with broad-spectrum anti-infective activity.

## Introduction

Resistance to antibiotics has become increasingly common among bacterial pathogens over the past few decades [1], [2]. For example, our resources to treat infections with extensively drug-resistant *Mycobacterium tuberculosis* are extremely limited and require a therapy based on a combination of different classes of antibiotics [3]. The emerging class of antibiotic-resistant bacteria, the carbapenem-resistant Enterobacteriaceae, which kills almost half of infected patients, is also a major health concern as all antibiotics currently available are ineffective [2]. Despite this trend, the antibacterial drug development pipeline flow is low and the number of new drugs available is rapidly decreasing [4], [5]. With notable increases in antibiotic resistance, the aging of the population and the fact that infectious diseases remain one of the leading causes of death worldwide, there is an urgent need for additional and diverse therapeutic strategies to treat infections [6].

Promising approaches for treatment of infectious diseases have been emerging. These include anti-virulence agents that target bacterial virulence determinants, or host-directed therapies, such as immunomodulatory drugs that enhance host immunity to promote more effective anti-microbial attack [7], [8], [9]. Host-targeted approaches possess major advantages compared to classic antibiotics that aim to kill or reduce bacterial growth, such as reducing selection for resistance genotypes, as there is less or no selective pressure directly imposed on the pathogen. Moreover, stimulation of the innate immune response may provide broad-spectrum protection against a range of pathogenic microorganisms, including bacteria, virus and parasites. Host-directed therapies may be used as adjunct treatments to synergize with commonly used anti-microbial drugs and may also allow diversification of therapeutic strategies currently available.

Protein ubiquitination is a reversible post-translational modification that regulates diverse cellular processes, such as DNA repair, cell division, signaling, protein degradation and notably, innate immune function. Ubiquitination occurs by covalent attachment of an ∼8.5 kDa ubiquitin molecule to a lysine residue in the target protein by the sequential action of three enzymes; a ubiquitin-activating enzyme (E1), a ubiquitin-conjugating enzyme (E2) and a ubiquitin-ligase enzyme (E3) [10]. Ubiquitin is removed from proteins by deubiquitinases (DUB) by proteolysis [11]. The human genome encodes over 100 proteins that possess putative DUB activity but physiological substrates of these proteins remain poorly defined for most [12]. DUB enzymes have established roles in a broad spectrum of diseases such as cancer, viral infection and neurodegenerative disorders [13], [14], [15]. Although the function of most DUBs in immune regulation is not known, a few are key players in the modulation of innate immunity and inflammation. For example, the deubiquitinases, A20 and CYLD, control NF-κB signaling, a critical pathway in immunity and cell survival [16], [17]. Control of ubiquitination also plays an established role in targeting invading pathogens for autophagic capture and degradation, and therefore presumably is subject to regulation by DUBs [18], [19]. Altering ubiquitination pathways may represent a way to modulate antibacterial autophagy and intracellular proliferation of pathogens.

The ubiquitin system and the DUB enzymes themselves have become a new class of interesting therapeutic targets [20], [21]. Although no DUB inhibitors are yet in clinical trials, diverse inhibitors have already been described, including the USP14 inhibitor IU1 [22] and inhibitors specific to USP7 [23], USP2 and UCH-L3 [24]. In addition, a small cell-permeable molecule, WP1130, also known as Desgrasyn, which selectively inhibits a subset of cellular DUBs, has been described recently as a potential anti-cancer therapeutic [25]. This molecule causes depletion of monomeric ubiquitin molecules and accumulation of ubiquitinated proteins in cells [26]. A previous study demonstrated that WP1130 directly inhibits *in vitro* activity of specific DUBs like USP9x, USP5, USP14 and UCH37, without affecting others, showing some degree of specificity [26]. However, the full-spectrum of WP1130 DUB targets as well as its mechanism of action are still unknown.

We previously found that WP1130 has anti-infective activity, reducing intracellular replication of some bacterial and viral pathogens. Replication of murine norovirus in a murine macrophage-like cell line, and other RNA viruses, were significantly reduced by WP1130 [27], [28]. This antiviral activity was at least in part mediated by inhibition of USP14, a proteasome associated DUB that also controls induction of the unfolded protein response. Moreover, we recently showed that DUB inhibition by WP1130 increases killing of the gram-positive bacterium, *Listeria monocytogenes,* within macrophages [29]. WP1130 treatment rapidly enhanced localization of the antimicrobial effector iNOS to the bacterial phagosome. These recent observations suggest that perturbation of protein ubiquination in host cells by small molecule DUB inhibitors may be an effective strategy to reduce infection that will cause minimal selective pressure on the pathogen itself.

*L. monocytogenes* and noroviruses, along with *Salmonella* and *Toxoplasma*, are among the leading causes of food and waterborne diarrheal diseases worldwide. In the US alone, food-borne pathogens are estimated to cause 9.4 million infections and around 1,350 deaths annually, and are associated with substantial healthcare costs [30]. However, there are limited vaccines or anti-microbial drugs available to prevent or treat these infections. As the DUB inhibitor WP1130 showed activity against two of these food-borne pathogens, exploiting the ubiquitin system for the development of a new class of broad-spectrum therapeutics to treat these infections is appealing. However, therapeutic use of WP1130 itself is limited due to its low solubility and poor bioavailability in animals [27]. Here we use *L. monocytogenes* as a model pathogen to screen a small library of WP1130-derivative molecules for anti-infective efficacy in macrophages, a major reservoir for many intracellular pathogens, with the overall goal of finding derivatives with better solubility and anti-infective activity.

## Materials and Methods

### Compounds

All small molecules were dissolved in DMSO (Sigma-Aldrich, St. Louis, MO), aliquoted and stored at −80°C. WP1130 and compounds 1 to 8 were synthesized by the Vahlteich Medicinal Chemistry Core (University of Michigan). Compound 9 was synthesized by Evotec (UK) Ltd, 114 Milton Park, Abingdon OX14 4SA, United Kingdom. Library numbers for each compound from the University of Michigan (CCG) and Evotec (EO) are: compound 1: CCG-206741; compound 2: CCG-208902; compound 3: CCG-206468; compound 4: CCG-206472; compound 5: CCG-206526; compound 6: CCG-206475; compound 7: CCG-206555; compound 8: CCG-208806; compound 9: EOAI3402143.

### Cell culture, bacterial strain and viruses

RAW264.7, a macrophage-like cell line, was used in this study because macrophages are a relevant major reservoir for many intracellular pathogens. Cells were originally obtained from American Type Culture Collection (ATCC; Manassas, VA) and were grown in Dulbecco’s modified Eagle medium (DMED) supplemented with 10% fetal bovine serum (FBS), 1% non-essential amino acids and 1% HEPES at 37°C with 5% CO_2_. HG23 cells containing a Norwalk virus replicon were developed by and obtained from Dr. Kyeong Chang (Kansas State University) and cultured as previously described [31]. *L. monocytogenes* 10403S was grown in brain heart infusion (BHI) broth statically at 30°C overnight [32]. The MNV-1 strain (GV/MNV1/2002/USA) MNV-1.CW3 was propagated and used at passage 6 for all experiments [33].

### *L. monocytogenes* intracellular growth assay

RAW264.7 cells were cultured at an initial density of 4×10^6^ cells per 24-well plate and allowed to adhere overnight. Macrophages were treated with 2.5 or 5 µM of DUB inhibitors dissolved in dimethyl sulfoxide (DMSO) for 0.5 or 1 h or with equivalent volume of DMSO (vehicle control) at 37°C. For dose-response curves, macrophages were incubated with 5, 2.5, 1 and 0.5 µM of compounds 1, 3 and 4 or 2.5, 2, 1 and 0.5 µM of compounds 2 and 9 for 0.5 h or 1 h at 37°C. DUB inhibitors were removed from cell culture prior to infection by changing the medium. Macrophages were infected with *L. monocytogenes* at a multiplicity of infection (MOI) of 1 for 0.5 h at 37°C. The inoculum was removed by washing infected cells three times with Dulbecco’s phosphate-buffered saline (PBS), and the cells were incubated in medium containing 10 µg/ml of gentamicin to kill extracellular bacteria for the duration of the experiment. At 8 h post-infection, cells were lysed in 1 ml 0.1% Triton X-100, serially diluted and plated on Luria-Bertani (LB) agar to enumerate the number of colony forming units (CFU). CFU were counted using the Acolyte SuperCount (Microbiology International, Frederick, MD) plate reader and software. Results were normalized to DMSO-treated cells.

### Bacterial growth curves

For bacterial growth curve in broth culture, an overnight culture of *L. monocytogenes* was back-diluted 1∶50 into BHI broth in the presence of 5 µM of compounds 1 to 4, 2.5 µM of compound 9 or equivalent volume of DMSO and incubated statically at 30°C for up to 8 h. At indicated time points (0, 2, 4, 6 and 8 h), a 50 µl-aliquot of bacteria was serially diluted and plated on LB agar to determine the number of CFU. To test the bactericidal properties of compound 9, an overnight culture of *L. monocytogenes* was back-diluted 1∶50 into BHI broth and allowed to grow at 37°C with shaking until an optical density at 600 nm (OD_600_) of ∼0.6–0.7 was obtained. Bacteria were then exposed to 1, 2.5 or 5 µM of compound 9 or equivalent volume of DMSO for 2 h at 4°C. After incubation, bacteria were serially diluted and plated on LB agar to determine CFU.

### Virus infection and plaque assay

RAW264.7 cells were plated at a density of 5×10^5^ cells/ml in each well of a 12-well plate and allowed to attach overnight. The following day, cells were incubated with either 5 µM WP1130 or 2.5 µM compound 9, for 30 min at 37°C. After incubation, cells were placed on ice for 1 hour where they were infected with MNV-1 at an MOI of 5. Cells were then washed three times with ice-cold PBS. Cells treated with compound 9 received media alone after washing, while media containing WP1130 or DMSO was added back to cells and the infection was allowed to proceed for 8 hours. Cells were freeze-thawed twice, and viral titers were determined by plaque assay as previously described [34].

### HuNoV replicon assay

HG23 cells containing the Norwalk virus replicon plasmid under G418-selection were plated at 5×10^5^ cells per well in 12-well plates and allowed to attach overnight. Afterwards, media was aspirated and replaced with fresh media containing DMSO, 5 µM WP1130, or 2.5 µM compound 9. DMSO and WP1130 remained on the cells for 24 hours, whereas compound 9 was removed after 2 hours. At the end of the experiment, media was aspirated, cells were washed once with PBS and total cellular RNA was isolated using the QIAGEN RNeasy Mini kit (QIAGEN, Valencia, CA). Following the manufacturer’s protocol, RNA was subjected to two rounds of DNase treatment. First, an on-column RNase-free DNase treatment (QIAGEN, Valencia, CA) was performed for 15 minutes at room temperature. After elution, another round of DNase treatment was performed using the DNA-free DNase removal kit (Ambion, Austin, TX). RNA concentration and purity were measured using a spectrophotometer, calculating the 260/280 ratio. RNA integrity (as well as the absence of DNA contamination) was confirmed by One-Step Reverse Transcription-Polymerase Chain Reaction (RT-PCR) using “No RT” controls. Norwalk virus genomes were then quantitated by qRT-PCR as previously described [31].

### Cell viability assay

For cell viability assays in conditions that mimicked the bacterial infection protocol, RAW264.7 cells were cultured at an initial density of 4×10^6^ cells per 96-well plate. Cells were treated with 2.5 or 5 µM DUB inhibitors dissolved in DMSO for 0.5 h or 1 h or with equivalent volume of DMSO. DUB inhibitors were removed after treatment by washing three times with PBS, and cells were incubated in culture media for 6.5 h at 37°C. The WST-1 reagent (Roche, San Francisco, CA), which measures mitochondrial dehydrogenase activity, was diluted 1 in 10 in media and cells were incubated at 37°C for an additional 1.5 h. The optical density at 440 nm (OD_440_) and 600 nm (OD_600_) was determined and results were normalized to DMSO-treated cells.

For cell viability assays in conditions that mimicked the viral infections, RAW264.7 cells were cultured as described above and treated for 0.5 h with 5 µM WP1130 or 2.5 µM compound 9, incubated for 1 h on ice, washed three times with ice-cold PBS and incubated for an additional 8 h in presence of DMSO and WP1130 only. One and a half hours prior to the end of the experiment, the WST-1 reagent was diluted 1 in 10 in the media. For cell viability in HG23 replicon cells, DMSO and WP1130 remained on the cells for 24 h before incubation with the WST-1 reagent, whereas compound 9 was removed after a 2 h exposure and the WST-1 reagent added after an additional 22 h.

### Protein extraction, SDS-PAGE and immunoblotting

RAW264.7 cells were cultured at an initial density of 4×10^6^ cells per 6-well plate and allowed to adhere overnight. Macrophages were treated with 2.5 µM compound 9 for the indicated period of time. To obtain whole-cell lysates, cells were lysed in cell lysis buffer (10 mM Tris pH 8, 150 mM NaCl, 1% NP-40, 10 mM EDTA pH 8, 1 mM DTT and 1X protease inhibitors), incubated for 15 min on ice and briefly sonicated. Samples were diluted in 5X concentrated sample buffer containing β-mercaptoethanol and denatured at 95°C for 10 min. The samples were separated by SDS-PAGE on an 8% acrylamide gel and transferred to polyvinyldene fluoride membrane (PVDF, Millipore). Immunodetection was performed using an anti-ubiquitin monoclonal antibody (P4D1 clone, Santa Cruz), according to manufacturer’s instructions.

### Statistical analysis

Results represent the means +/− standard error for at least three independent experiments performed in duplicate or triplicate. Statistical analysis was performed using GraphPad Prism software. The two-tailed student t test or the one-way analysis of variance (ANOVA) were used to determined statistical difference. Dunnett or Bonferroni post-tests were used after a significant ANOVA to compare the different groups and determine which differences were significant. *p<0.05, **p<0.01, ***p<0.001. For EC50 determination, non-linear regression analysis was performed using GraphPad Prism software.

## Results and Discussion

### Determination of the cellular toxicity of the WP1130-derivatives compounds

**Figure 1:**
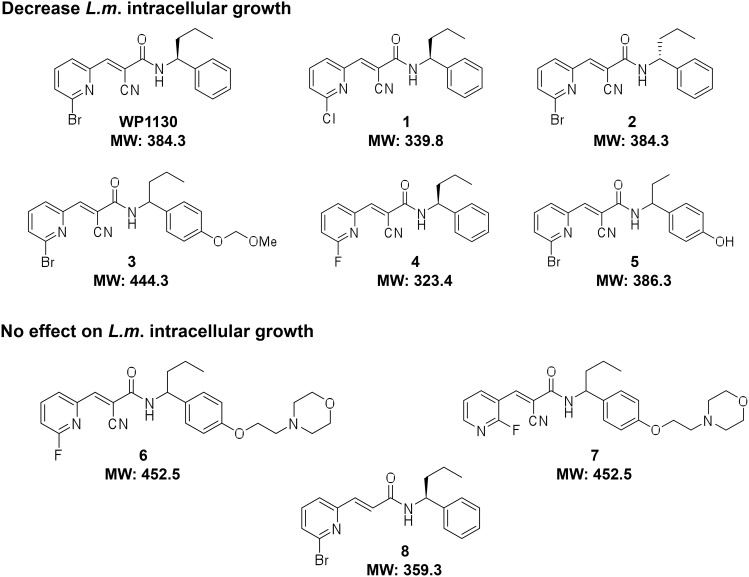
Structures and molecular weights of WP1130-derivative compounds.

**Figure 2:**
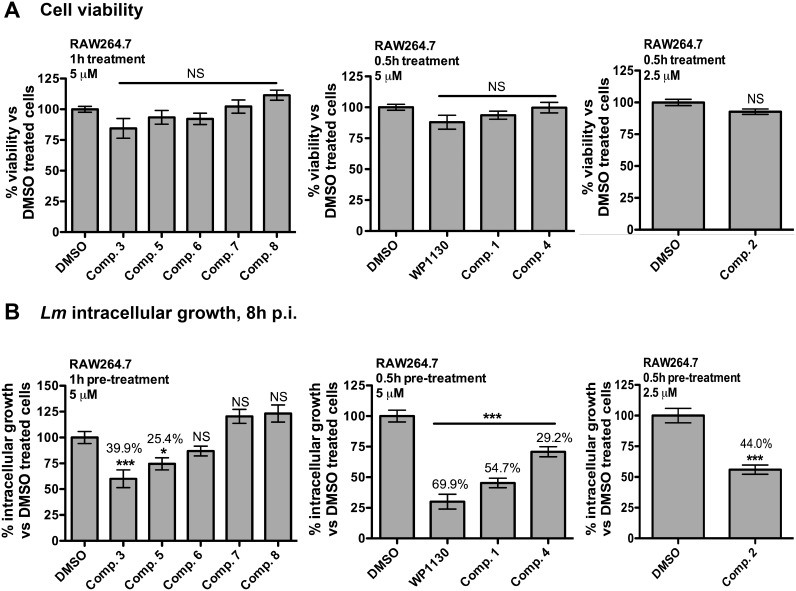
Selected compounds have anti-infective activity against *L. monocytogenes* intracellular replication with minimal cellular toxicity. **A**. RAW264.7 macrophages were incubated at 37°C for 0.5 h or 1 h with DMSO (equivalent volume) or the indicated concentration of WP1130 or derivatives. After incubation, cells were washed and incubated for an additional 8 h before performing the WST-1 assay for cell viability. Results represent the percent viability compared to the DMSO-treated cells from three independent experiments. **B**. RAW264.7 cells were incubated for the indicated time with DMSO (equivalent volume) or the indicated concentration of WP1130 or WP1130-derivatives. After incubation, the medium was removed, and cells were infected at 37°C for 0.5 h at an MOI of 1 with *L. monocytogenes* in the absence of additional drugs. Following the 0.5 h infection, cells were washed and gentamycin (10 µg/ml) was added to kill extracellular bacteria. Intracellular bacteria were enumerated at 8 h post-infection. The data represent percent of *Listeria* intracellular growth compared to DMSO-treated cells from three independent experiments. Significant differences were calculated using one-way ANOVA and Dunnett’s post-test (NS, not significant, *p<0.05, ***p<0.001).

We initially aimed to test a small library of 28 rationally-designed molecules based on the structure of the parent compound, WP1130, synthesized at the University of Michigan Vahlteich Medicinal Chemistry Core (Fig. 1 and data not shown). We first tested these compounds for cellular toxicity in our model host cell line, RAW267.4 macrophages, in conditions that mimic our *L. monocytogenes* infection assay. Cells were pre-treated with the indicated concentrations of compounds for 0.5 or 1 h or with equivalent volume of DMSO, washed and then incubated for an additional 8 h before measuring cell viability. Fig. 2A showed results for the subset of compounds selected for this study, depicted in Fig. 1. Most of the compounds, including compounds 3, 5, 6, 7 and 8 caused no significant toxicity when used at 5 µM for 1 h of pre-treatment, whereas 0.5 h pre-treatment with 5 µM was optimal for compounds 1 and 4. Compound 2 exhibited a slight increase in toxicity, so we used a working concentration of 2.5 µM for 0.5 h of pre-treatment. Based on these results, we used conditions for pre-treatment with each derivative that minimized cellular toxicity.

### Effect of the WP1130-derivatives on *L. monocytogenes* intracellular growth

In order to identify new WP1130 derivatives with anti-infective activity, we used the Gram-positive bacterial human pathogen *L. monocytogenes* as a model infection system. *L. monocytogenes* is taken up by macrophages into a membrane-bound phagosome, and rapidly escapes this compartment by secretion of a pore-forming toxin, listeriolysin O, and bacterial phospholipases [35], [36]. Replication of *L. monocytogenes* occurs in the cytoplasm of the host cell and actin polymerization at the bacterial surface propels some bacteria into neighboring cells without exposure to the extracellular environment. Using the conditions indicated by our cell viability assays, we screened the library of small compounds for effects on *Listeria* intracellular growth. We used a previously described infection protocol [29], where cells were pre-treated with compound before a 30 min infection in the absence of drug. The number of intracellular colony forming units (CFU) was determined at 8 h post-infection. We chose to only pre-treat cells with compound before performing the infection in order to minimize any direct effect of the compounds on the bacteria. Moreover, based on our previous observations with the parent compound, the anti-infective effect of WP1130 occurs at an early stage of infection [29]. Among the 28 compounds used, only 5 derivatives caused a significant decrease in *Listeria* intracellular viability, and were selected for further analysis (Fig. 2B). The original WP1130 compound remained the most effective, causing a decrease in *Listeria* intracellular viability of 69.9%, followed by compounds 1 through 5 (decreased *Listeria* intracellular viability by 54.7%, 44%, 39.9%, 29.2% and 25.4% respectively). Compounds 6, 7 and 8 are shown as examples of derivatives that had no significant effect on *Listeria* intracellular growth. To evaluate potency of these new compounds, we also performed dose-response curves for the four best WP1130 derivatives with varying concentrations ranging from 0.5 µM to 2.5 µM for compound 2 and 0.5 µM to 5 µM for compounds 1, 3 and 4 (Fig. S1). The EC50 for each compound, which represents drug concentration that gives half-maximal response, are shown in Table 1. For compound 3, all concentrations above 5 µM did not result in decrease of intracellular *Listeria* viability, explaining why no EC50 was determined. Notably, compounds with the lowest EC50 values were also the most effective for decreasing intracellular *Listeria* viability at our working concentration.

**Table 1:** EC50 and percentage decrease in intracellular *Listeria* viability for effective compounds.

Compound	EC50 (µM)^a^	Decrease intracellular *listeria* viability (%)^c^
Comp. 1	1.10	54.7
Comp. 2	1.76	44.0
Comp. 3	N/A^b^	39.9
Comp. 4	2.88	29.2
Comp. 9	1.92	66.1

a EC50 were determined by scoring bacterial intracellular growth in cells treated with varying concentrations of compounds and then infected with *Listeria*.

b N/A: not applicable.

c The percentages of growth inhibition were determined using conditions shown in Figure 2B and Figure 5B, which represent the maximal effective drug concentration used.

### Effects of the WP1130-derivatives on *L. monocytogenes* growth in broth culture

**Figure 3:**
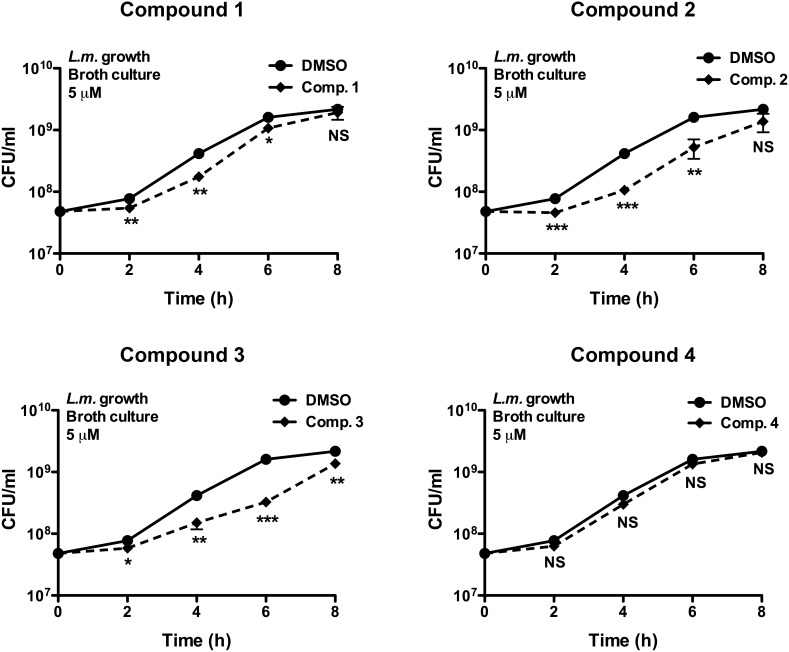
Effect of selected compounds on *in vitro* growth of *L. monocytogenes* in broth culture. An overnight culture of *L. monocytogenes* was back-diluted into BHI broth media in the presence of 5 µM of the indicated compound or equivalent volume of DMSO. At different time points during growth at 37°C, bacteria were plated on LB agar, and the number of colony forming units (CFU) was determined. Results represent the mean of three independent experiments. Significant differences were calculated using two-tailed student’s t test (NS, not significant, *p<0.05, **p<0.01, ***p<0.001).

We showed previously that WP1130 had no effect on *Listeria* growth in broth culture in the absence of host cells, suggesting that the decrease in intracellular bacterial viability was a consequence of WP1130 on target cells [29]. To determine whether the new WP1130-derivatives acted similarly, we tested the four best WP1130 derivatives for their effect on *Listeria* growth in axenic culture (Fig. 3). Compound 4 had no effect on *Listeria in vitro* growth in the absence of host cells. Compounds 1 and 2 caused a slight decrease in growth rate without affecting the total number of colony forming unit (CFU) per ml by 8 h. Finally, compound 3 caused a slight decrease in the *in vitro* growth of *Listeria*. While we cannot rule out the possibility that direct anti-bacterial activity of these compounds against intracellular bacteria contributes modestly to their overall anti-infective efficacy, taken together, our data suggest that compounds 1, 2 and 4, as for the parent compound WP1130, largely confer anti-infective activity by acting on host cell targets.

### Design of a new derivative with optimized chemistry

As shown above, five new WP1130 derivatives showed anti-infective activity. However, the decrease in *Listeria* intracellular growth was not as pronounced as for the parent WP1130 compound. We next aimed to design a new molecule based on the information gained from the structural characteristics of the compounds screened above. DUB inhibition by WP1130 likely occurs at least in part through covalent modification of the active site cysteine residues, due to the α, β-unsaturated carbonyl group of WP1130. In agreement with this hypothesis, compound 8, where the cyano group had been removed, showed no activity in our *L. monocytogenes* infection assay. We also tried WP1130’s enantiomer (compound 2), however, the anti-infective activity of this compound against *Listeria* intracellular growth was not improved by the modification. We found that the halogen group attached to the distal pyridyl ring was important for the anti-infective activity of WP1130 and derivatives against intracellular *L. monocytogenes*. Exchange of the bromide moiety for chloride (compound 1) only had a minor effect on compound activity whereas a fluoride substitution (compounds 4 and 6) caused a partial or complete loss of efficacy. The distal right benzene ring was modified by addition of a polar side chain to increase solubility and bioavailability of the WP1130 derivatives. A small decrease in anti-infective efficacy was observed with a smaller polar side chain (compounds 3 and 5) whereas addition of larger side chains caused a complete loss of activity of the derivatives (compounds 6 and 7). It should be noted, however, that in addition to a larger side chain, the configuration of the distal pyridyl ring was also modified and the halogen group was replaced in compound 7. The complete loss of activity for this specific compound may have been caused by these multiple changes. However, the specific effect of these modifications on overall DUB inhibition is unknown as these substitutions may influence interactions between the derivatives and a subset of DUBs (either decreasing affinity for all DUB targets or changing the target specificity of the compound) or by increasing the binding affinity for other cellular targets that are not relevant for the anti-infective activity.

**Figure 4:**
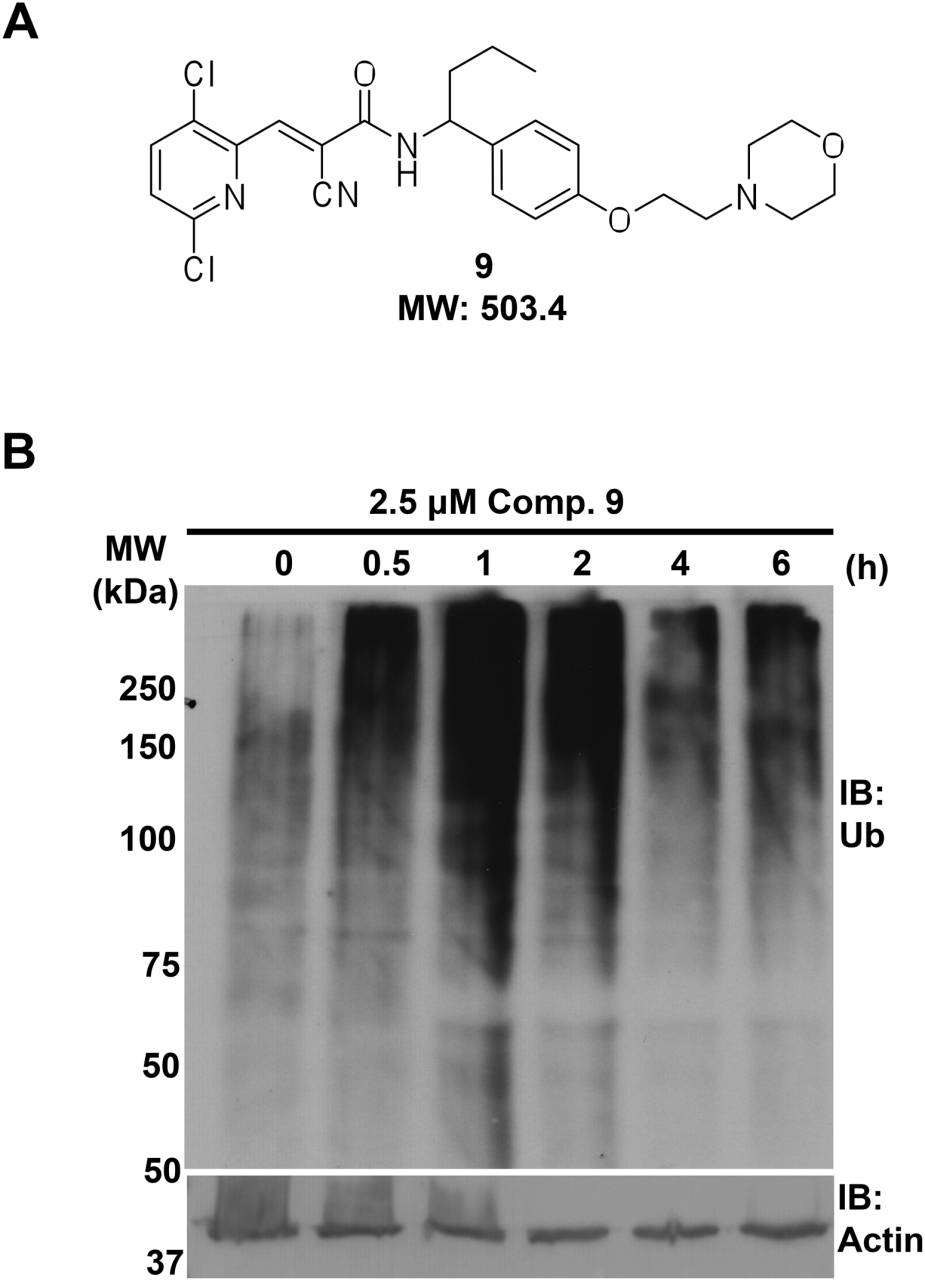
Inhibition of DUB activity by compound 9 in RAW264.7 macrophages. **A**. Structure and molecular weight of compound 9. **B**. Uninfected RAW264.7 cells were treated with 2.5 µM of compound 9 for the indicated time. Whole-cell lysates were analyzed by SDS-PAGE and immunoblotting with antiserum against ubiquitin and ubiquitinated proteins (Ub) or against actin (loading control).

A new compound was generated through rational design based on the structural and functional characteristics observed with the initial series of WP1130 derivatives, with the aim of increasing solubility with new polar side chain substitutions and notably, with a double halogen group on the distal pyridyl ring to increase anti-infective efficacy, as depicted in Fig. 4A. Two chloride halogen groups replaced the original bromide on the pyridyl ring of WP1130 and a new polar side chain was added to the distal right benzene ring. The aqueous kinetic solubility for compound 9 is approximately 20 µM compared to 3.2 µM for WP1130, showing a >6-fold improvement in solubility.

To determine if compound 9 exhibited DUB-inhibitor activity like the parent WP1130 compound, we treated RAW264.7 cells with 2.5 µM compound 9 for the indicated time before extracting proteins and probing with an antibody that recognizes ubiquitin, mono-ubiquitinated and poly-ubiquitinated proteins. As shown in Fig. 4B, ubiquitinated proteins accumulated as soon as 0.5 h after addition of compound 9, and the effect lasted for at least 6 h of treatment. Similar results were obtained with cells treated only for 0.5 h with 2.5 µM of compound 9 before removal of the compound. Using the 0.5 h treatment protocol, an increase in total ubiquitinated proteins was observed up to 3.5 h after removal of the drug, with maximum detection of ubiquinitated protein at 1.5 h (data not shown). Moreover, compound 9 possessed strong inhibitory activity in a cell-based assay against a specific target DUB, USP9x, with an IC50 of 1.8 µM compared to 6.6 µM for WP1130 (Donato, N. J., manuscript in preparation). These results are consistent with compound 9 acting as a DUB inhibitor, and demonstrate that 0.5 h treatment is sufficient to significantly increase the cellular pool of ubiquitinated proteins.

### Compound 9 treatment limits *L. monocytogenes* intracellular growth in macrophages

**Figure 5:**
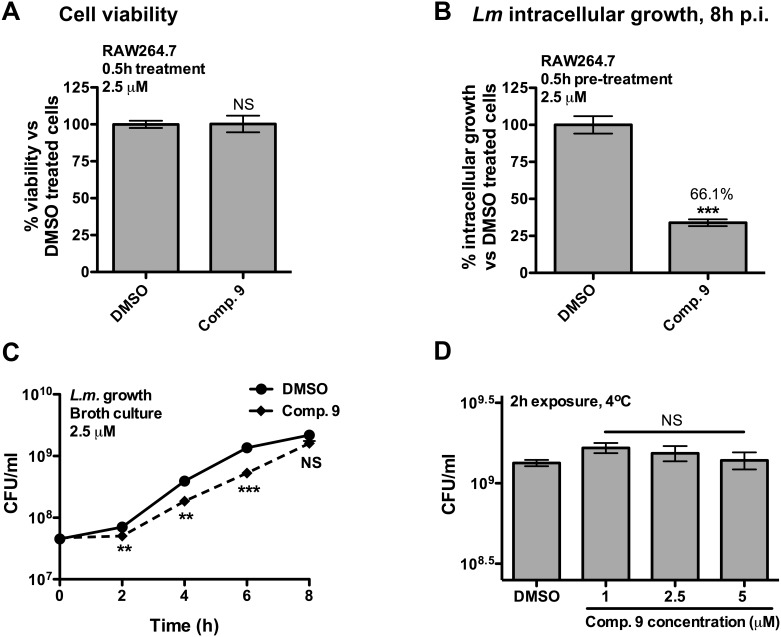
Anti-infective activity of compound 9 against *L. monocytogenes*. **A**. RAW264.7 macrophages were incubated at 37°C for 0.5 h with DMSO (equivalent volume) or 2.5 µM of compound 9. After incubation, cells were washed and incubated at 37°C for an additional 8 h before performing the WST-1 assay for cell viability. Results represent percent viability compared to the DMSO-treated cells from three independent experiments. **B**. RAW264.7 cells were incubated for 0.5 h with DMSO (equivalent volume) or 2.5 µM of compound 9. After incubation, the medium was removed, and cells were infected for 0.5 h at an MOI of 1 with *L. monocytogenes*. Following the 0.5 h infection cells were washed and gentamycin (10 µg/ml) was added to kill extracellular bacteria. Intracellular bacteria were enumerated at 8 h post-infection. The data represent percent of *Listeria* intracellular growth compared to DMSO-treated cells from three independent experiments. **C**. An overnight culture of *L. monocytogenes* was back diluted into BHI broth media in the presence of 2.5 µM of compound 9 or equivalent volume of DMSO. At different time points, bacteria were plated on LB agar to determine the number of colony forming units (CFU). Results represent the mean of three independent experiments. **D**. *L. monocytogenes* in exponential growth phase were exposed to the indicated concentration of compound 9 for 2 h at 4°C. After incubation, bacteria were plated on LB agar and the number of CFU was determined. Significant differences were calculated using two-tailed student’s t test (A and B) or a one-way ANOVA and Dunnett’s post-test (C and D) (NS, not significant, **p<0.01, ***p<0.001).

As treatment of 0.5 h with 2.5 µM of compound 9 significantly increased the level of cellular ubiquitinated proteins, we used this condition to test cellular toxicity of compound 9 and its effect on *Listeria* intracellular growth. As shown in Fig. 5A, little cellular toxicity was observed in RAW264.7 cells treated with compound 9. Notably, compound 9 promoted anti-infective efficacy, decreasing *Listeria* intracellular viability of 66.1% by 8 h post-infection, close to the efficacy observed for WP1130 treatment (69.9%) (Fig. 5B). To test whether the observed effect of compound 9 on intracellular growth of *Listeria* was due to DUB activity, and not to direct effects of the compound on bacterial growth or viability, we performed a growth curve in broth culture in the presence of compound 9. As shown in Fig. 5C, 2.5 µM of compound 9 caused a modest decrease in the growth rate but did not significantly affect overall CFU per ml by 8 h of growth in axenic broth culture. To test more directly whether compound 9 had bactericidal properties, we exposed bacteria in exponential growth phase to different concentrations of compound 9, and incubated them for 2 hours at 4°C in order to limit further bacterial growth. No bactericidal activity was observed for concentrations of compound 9 up to 5 µM (Fig. 5D). These results suggest that compound 9 likely acts as an anti-infective compound by perturbing biological processes within the host macrophage.

### Compound 9 has anti-infective activity against murine and human norovirus

**Figure 6:**
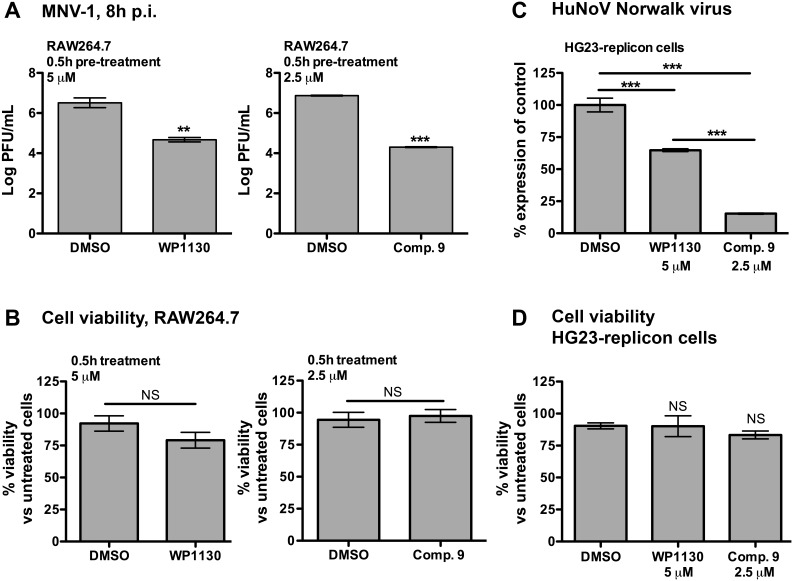
Anti-infective activity of compound 9 against murine and human norovirus. **A**. RAW264.7 cells were incubated at 37°C with DMSO (volume-matched), 2.5 µM compound 9 (right graph) or 5 µM WP1130 (left graph) for 0.5 h prior to infection. After incubation, cells were infected with MNV-1 (MOI 5) in the presence of compounds or DMSO for 1 h on ice. Cells were washed and only DMSO and WP1130 were added back to the cells. Infection proceeded for 8 h. Virus titers were determined by plaque assay. **B**. RAW264.7 macrophages were incubated for 0.5 h with DMSO (volume-matched), 2.5 µM compound 9 (right graph) or 5 µM WP1130 (left graph) at 37°C, followed by 1 h of incubation on ice. Cells were then washed and incubated for an additional 8 h before performing the WST-1 assay for cell viability. Results represent the percent of viability compared to the untreated cells. **C**. HG23-replicon cells were incubated for 24 h with DMSO or 5 µM WP1130. Alternatively, cells were incubated for 2 h with 2.5 µM compound 9 and washed. After 24 h incubation, Norwalk virus genomes were quantitated by qRT-PCR. Norwalk virus genome copy number was normalized to DMSO control. **D**. HG-23 replicon cells were incubated as described in C before performing the WST-1 cell viability assay. Results represent the percent of viability compared to the untreated cells. Results represent the mean of three independent experiments and significant differences were calculated using two-tailed student’s t test (A and B) or one-way ANOVA and Bonferroni’s post-test (C and D) (**p<0.01, ***p<0.001, NS, not significant).

Previous work showed that the parental compound WP1130, is effective against a wide-range of RNA viruses, including murine norovirus (MNV-1), a surrogate for human norovirus (HuNoV) [27], an important foodborne pathogen and one of the leading causes of non-bacterial gastroenteritis worldwide [30], [37]. Pre-treatment of RAW264.7 cells with WP1130 resulted in an average 2.0-log decrease in MNV-1 titers compared to the DMSO vehicle control [27]. To test whether the anti-infective activity of compound 9 could affect viral replication, we tested its effect on the replication of MNV-1. When RAW264.7 cells were pre-treated for 0.5 h with 2.5 µM of compound 9, half the working concentration necessary for WP1130’s antiviral activity, MNV-1 titers decreased more than 2.5-logs compared to DMSO-treated cells (Fig. 6A). No significant toxicity was observed at this concentration of compound 9 in conditions that mimicked the MNV-1 infection protocol (Fig. 6B).

We also assessed whether compound 9 was effective against the HuNoV Norwalk virus. As it is currently not possible to follow a complete infectious cycle of HuNoV *in vitro* [38], [39], we took advantage of the Norwalk virus replicon system, which allows the measurement of Norwalk virus genomic replication in a tissue culture system [31]. Replicon-bearing hepatoma cells (HG23) were grown in the presence of 2.5 µM of compound 9 for 2 hours or 5 µM WP1130 for 24 hours, after which Norwalk virus genomes were quantitated using qRT-PCR as previously described [31]. Compound 9 significantly reduced the level of detectable Norwalk virus genomes by 84.7% (Fig. 6C). Again, no toxicity was observed at this concentration of compound 9 in conditions that mimicked the infection protocol (Fig. 6D). These results demonstrate that at the concentration tested this compound significantly inhibited both MNV-1 infection and Norwalk virus replication *in vitro* without affecting cell viability.

### Conclusion

By testing a small library of rationally-designed molecules based on the structure of the parent deubiquitinase inhibitor, WP1130, we have identified new compounds that show *in vitro* anti-infective activity, and found one lead candidate, compound 9, that is effective against diverse microorganisms, including *L. monocytogenes* and norovirus, two important foodborne pathogens. This compound is more soluble than the original WP1130 molecule and possesses anti-infective activity at lower concentration, suggesting improved potential for use as an anti-infective drug.

With increased emergence of antibiotic resistance, alternative strategies to fight infections are urgently required. Host-targeted therapies that enhance immunity to promote effective anti-microbial responses will present additional options for treatment [7], [8]. DUB enzymes are promising candidates for development of such immunomodulatory drugs, as they are key players regulating pathways that control immunity and inflammation [16]. However, the specific spectrum of DUB enzymes targeted in macrophages by compound 9 remains to be determined. Learning more about cellular DUB targets of compound 9 that are critical for anti-infective function will aid in directing iterative synthesis of novel structural variants with increased target specificity and anti-infective activity. Moreover, compound 9 may be a useful probe to increase our knowledge of a family of poorly characterized enzymes, the cellular deubiquitinases, and their functions in cell biology and immunity.

In summary, small molecule compounds that target host functions to modulate the cellular response to infection, like the compound 9 DUB inhibitor described here, may represent a platform for development of new broad-spectrum anti-infective drugs.

## Supporting Information

Figure S1 **Dose-response curves for selected compounds.** RAW264.7 cells were incubated with DMSO (equivalent volume) or the varying concentrations of WP1130-derivatives for 0.5 h (compounds 1, 2, 4 and 9) or 1 h (compound 3). After incubation, the medium was removed, and cells were infected at 37°C for 0.5 h at an MOI of 1 with *L. monocytogenes* in the absence of additional drugs. Following the 0.5 h infection, cells were washed and gentamycin (10 µg/ml) was added to kill extracellular bacteria. Intracellular bacteria were enumerated at 8 h post-infection. The data represent percent of *Listeria* intracellular growth compared to DMSO-treated cells from three independent experiments performed in triplicate. Non-linear regression curves were performed using the GraphPad Prism software.
(TIF)
