# Supplementary figures and images for: Small Molecule Deubiquitinase Inhibitors Promote Macrophage Anti-Infective Capacity
Source: PLoS One. 2014 Aug 5;9(8):e104096. doi: 10.1371/journal.pone.0104096 (PMC4122495; doi:10.1371/journal.pone.0104096)

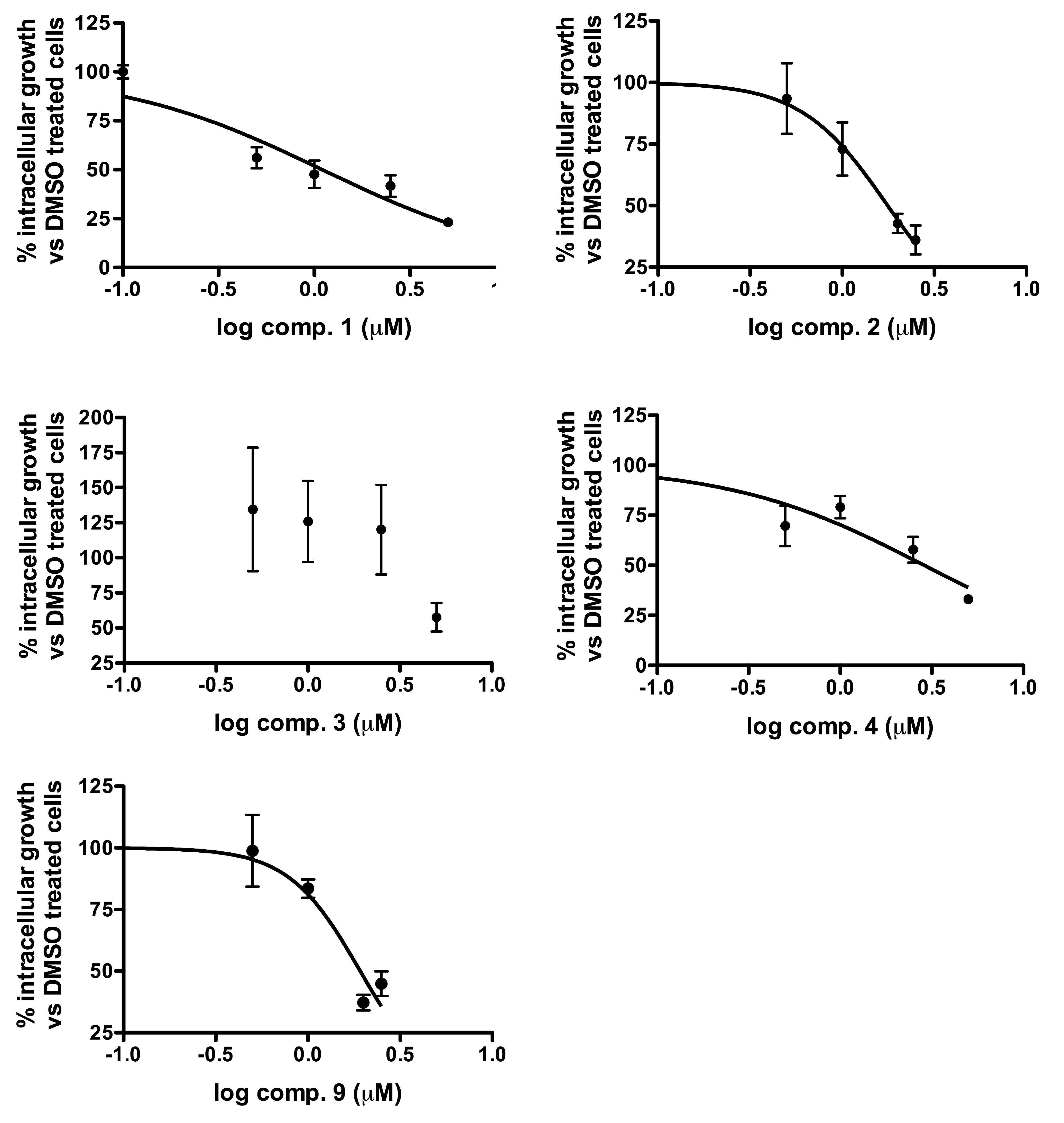

Supplement: Figure S1 — Dose-response curves for selected compounds. RAW264.7 cells were incubated with DMSO (equivalent volume) or the varying concentrations of WP1130-derivatives for 0.5 h (compounds 1, 2, 4 and 9) or 1 h (compound 3). After incubation, the medium was removed, and cells were infected at 37°C for 0.5 h at an MOI of 1 with L. monocytogenes in the absence of additional drugs. Following the 0.5 h infection, cells were washed and gentamycin (10 µg/ml) was added to kill extracellular bacteria. Intracellular bacteria were enumerated at 8 h post-infection. The data represent percent of Listeria intracellular growth compared to DMSO-treated cells from three independent experiments performed in triplicate. Non-linear regression curves were performed using the GraphPad Prism software. (TIF) [file pone.0104096.s001.tif]
